# Diagnostic Performance of ACR TI-RADS and ATA Guidelines in the Prediction of Thyroid Malignancy: A Prospective Single Tertiary Center Study and Literature Review

**DOI:** 10.3390/diagnostics13182972

**Published:** 2023-09-18

**Authors:** Ashkan Torshizian, Fatemeh Hashemi, Nastaran Khoshhal, Alireza Ghodsi, Houra Rastegar, Zohreh Mousavi, Maliheh Dadgar Moghadam, Masoud Mohebbi

**Affiliations:** 1 Student Research Committee, Faculty of Medicine, Mashhad University of Medical Sciences, Mashhad 13944-91388, Iran; 2 Metabolic Syndrome Research Center, Mashhad University of Medical Sciences, Mashhad 13944-91388, Iran; 3 Clinical Research Development Unit, Faculty of Medicine, Mashhad University of Medical Sciences, Mashhad 13944-91388, Iran; 4 Faculty of Medicine, Mashhad University of Medical Sciences, Azadi Sq., Mashhad 13944-91388, Iran

**Keywords:** thyroid nodule, malignancy, ACR TI-RADS, ATA guidelines

## Abstract

Aim: This study sought to compare two common risk stratification systems in terms of their diagnostic performance for the evaluation of thyroid malignancy. Methods: The American College of Radiology (ACR) Thyroid Imaging, Reporting and Data System (TI-RADS) and the American Thyroid Association (ATA) guidelines were compared among 571 thyroid nodules with definitive fine needle aspiration (FNA) cytology or postoperative histopathology. Ultrasound characteristics such as composition, echogenicity, shape, margin, size, and vascularity were assessed for each thyroid nodule. Diagnostic performance measures were determined and compared through receiver operating characteristic (ROC) curves, and decision curve analysis (DCA). Results: Of 571 nodules, 65 (11.4%) were malignant. The AUC, sensitivity, specificity, positive predictive value, and negative predictive value were 0.691, 49.2%, 84.9%, 29.6%, and 92.8% for ATA guideline, and 0.776, 72.3%, 79.2%, 30.9%, and 95.7%, for ACR TI-RADS, respectively. ACR TI-RADS was more sensitive (*p* = 0.003), while the ATA guideline was more specific (*p* < 0.001). DCA demonstrated that the ACR TI-RADS provided a greater net benefit than the ATA guideline. In addition, the net reduction in unnecessary biopsies is higher for ACR TI-RADS than ATA guidelines. The total number of indicated biopsies and unnecessary FNA rates were lower in ACR TI-RADS compared to ATA guideline (293 vs. 527 and 80.2 vs. 87.8). ACR TI-RADS presented no biopsy indication in seven malignant nodules (all categorized as TR2), whereas ATA guideline missed one. Hypoechogenicity was the most significant predictor of malignancy (OR = 8.34, 95% CI: 3.75–19.45), followed by a taller-than-wide shape (OR = 6.73, 95% CI: 3.07–14.77). Conclusions: Our findings suggest that each system has particular advantages in the evaluation of thyroid nodules. ACR TI-RADS reduces unnecessary FNA rates, however, malignant nodules categorized as TR2 might be missed using this system. Further evaluation of this group of nodules using Doppler and other ultrasound modalities is recommended.

## 1. Introduction

Thyroid nodules are a common disorder of the thyroid gland, with a significant increase in incidence over the last few decades [1]. The reported prevalence of thyroid nodules among the normal population varies depending on the detection method. Ultrasonography (US) and autopsy examination have shown prevalence rates of 19–35% and 8–65%, respectively [2]. The majority of thyroid nodules are detected incidentally in US; given their low malignancy rate (7–15%), it is crucial to avoid unnecessary biopsies [2,3].

Various guidelines recommend US as the first-line technique in the diagnosis and management of thyroid nodules as it is more sensitive than physical examination in the detection of thyroid nodules [3,4,5]. In 2017, the American College of Radiology (ACR) proposed a stratification system for US assessment of thyroid nodules to guide physicians in further diagnostic approaches and to reduce unnecessary fine needle aspiration (FNA) biopsy samplings [6].

The ACR Thyroid Imaging, Reporting and Data System (TI-RADS) categorizes nodules into five categories with increasing probability of malignancy from benign (TR1) to highly suspicious (TR5) [6]. The American Thyroid Association (ATA) also published a comprehensive guideline on the management of thyroid nodules in 2015. This guideline contains recommendations for serum marker evaluation, choice of imaging modalities, and categorization of thyroid nodules based on their US characteristics [3]. Premised on their sonographic pattern, the ATA guideline classifies thyroid nodules into five categories, ranging from benign with less than 1% risk of malignancy to highly suspicious with more than 70–90% likelihood of malignancy. The ATA guideline also indicates FNA recommendations based on nodules’ sonographic patterns and their size [3].

Several studies have compared ATA guidelines and the ACR TI-RADS to investigate how effectively they predict the presence of malignancy and how efficient they are at minimizing FNA biopsies [7,8,9,10]. However, these studies have employed distinct methodologies and achieved contradictory results [11].

To the best of our knowledge, no previous study has compared these two guidelines in the Iranian population. This study aimed to evaluate and compare the diagnostic performance of two commonly used thyroid US guidelines (ATA guideline and ACR TI-RADS) and compare their efficacy in reducing unnecessary FNA biopsy in patients referred for FNA sampling to a tertiary hospital in Mashhad, Iran. In addition, we assessed the effect of nodule size on the performance of these two classification systems.

## 2. Materials and Methods

### 2.1. Patient Selection

**Figure 1:**
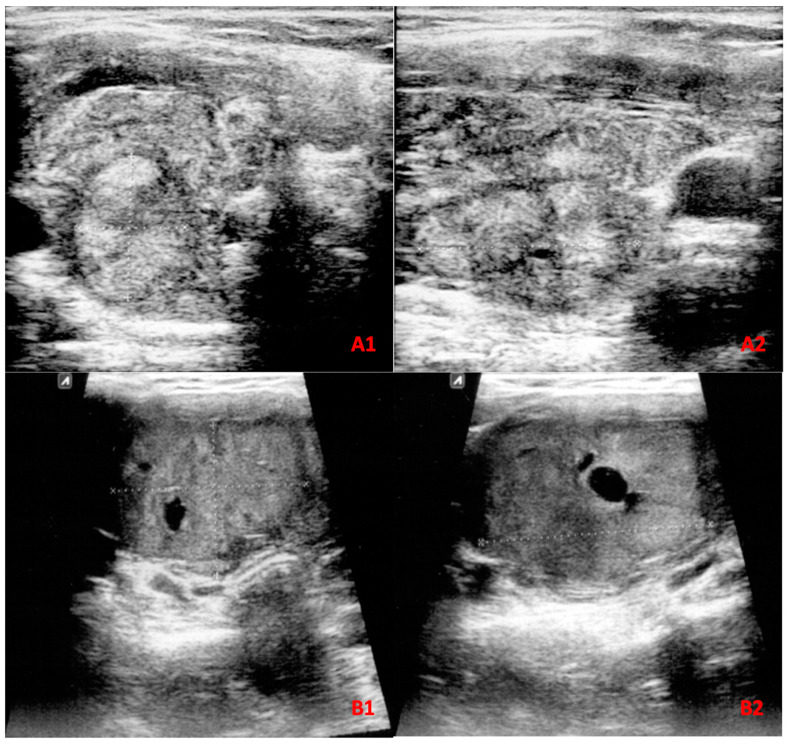
Transverse (**A1**) and longitudinal (**A2**) US views of a solid, isoechoic nodule with a smooth margin, largest diameter of 1.64 cm, and a taller-than-wide shape in a 41-year-old female. This nodule is classified as TR4 despite not being classifiable according to ATA guidelines. The nodule was finally diagnosed as FTC. Transverse (**B1**) and longitudinal (**B2**) US views of a nodule from a 31-year-old female patient illustrate a solid, isoechoic nodule with a smooth margin, largest diameter of 4.06 cm, and a wider-than-tall shape. This nodule is classified as TR3 and low suspicion by ACR TI-RADS and ATA guidelines, respectively. FNA cytology reported grade V Bethesda, and upon follow-up, the lesion was diagnosed as PTC by histopathology.

**Figure 2:**
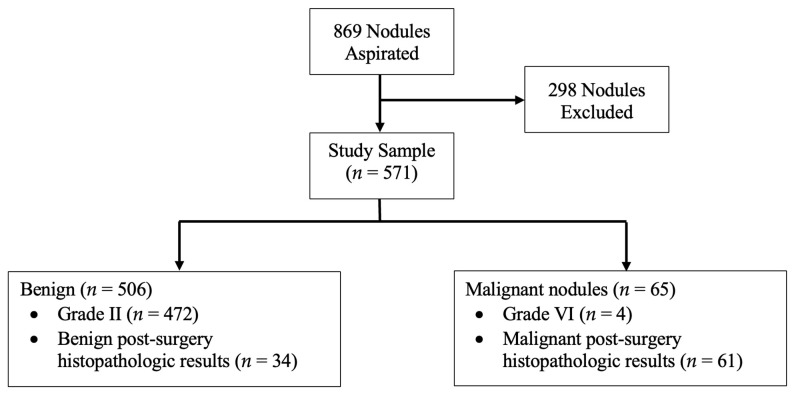
Study flowchart.

This prospective study was carried out on all patients with thyroid nodule(s) who underwent FNA biopsy from January 2018 to January 2022 at the Endocrinology Department of Imam Reza Hospital, affiliated with Mashhad University of Medical Sciences, Mashhad, Iran. During the study period, 813 patients with 869 aspirated nodules were referred to our department. These patients were followed for 6 months, and post-surgery histopathology results were recorded when available. Among all nodules, 283 nodules were excluded due to inconclusive final diagnosis, and 15 nodules were excluded as they were not classifiable according to the ATA guideline; however, they were assessed with ACR TI-RADS and followed to obtain a final diagnosis when available (Figure 1(A1,A2)). Of excluded nodules, 113 were classified as Bethesda grades III, IV, and V, while 170 nodules had nondiagnostic or unsatisfactory FNA findings (Bethesda grade I). As these patients either refused to undergo repeated FNA cytopathology examination or had a second inconclusive FNA cytopathology result and declined surgery within the 6-month follow-up period, a final diagnosis could not be obtained, and these nodules were thus excluded from further analysis. Eventually, 571 thyroid nodules were included of which 95 were diagnosed using histopathology results and 476 were diagnosed based on FNA cytology reports (Figure 2). Among nodules diagnosed as malignant, 61 out of 65 lesions had a confirmed post-surgical histopathologic result. Malignancy was diagnosed on the basis of Bethesda grade VI in four patients as three were lost to follow-up and the other refused surgery.

### 2.2. US Assessment

Thyroid US examination was performed using an 8 to 17 MHz linear probe (E-CUBE 15 EX, Alpinion Medical Systems Co., Ltd., Anyang, Republic of Korea) in most cases, as it provided excellent resolution and was suitable for detecting small nodules as well as subtle changes in the thyroid tissue. Nodules’ vascularity was also assessed using a high-frequency linear probe in color Doppler mode. However, in certain cases where specific nodules required better visualization or were located deep in the tissue, a curved probe with a lower frequency (1–4 MHz) was used as determined by the in-charge radiologist. The examination was performed in both transverse and longitudinal planes to obtain a comprehensive evaluation of the thyroid gland and nodules. The US characteristics of the nodules (nodule composition, echogenicity, vascularity, shape, margin, and presence of echogenic foci), and pathologic lymph nodes were recorded in an institutional checklist. Prior to FNA biopsy, two expert radiologists calculated ACR TI-RADS scores and categorized patients according to ATA guidelines independently. A radiologist with 15 years of thyroid US examination experience recalculated the scores during an FNA intra-procedural US examination in the event that there was a discrepancy between the reports of the two radiologists. 

### 2.3. FNA Assessment

FNA samples were collected using a 10 mL syringe and a 23-gauge needle under real-time US. Samples were smeared on a microscopic slide and fixated with 95% ethanol.

A pathologist interpreted FNA cytology in accordance with the Bethesda system for thyroid malignancy screening. Bethesda grade II was regarded as benign whereas grade VI was deemed malignant. Nodules with indeterminate FNA results (Bethesda grades I, III, IV, and V) were excluded unless their post-surgery histopathologic results or repeated FNA biopsy cytology reports led to a definitive diagnosis at 6-month follow-up (Figure 1). Regarding the benignity of nodules categorized as Bethesda grade II, it is worth mentioning that large prospective cohorts and metanalysis studies of the available literature have indicated a less than 3% risk of malignancy in this category of nodules [12]. Nevertheless, we followed patients with nodules categorized as Bethesda grade II for 6 months and at least one repeated US examination was made to assess these nodules for either rapid growth or extrathyroidal extension. However, no such cases were observed upon completion of our study and thus, a second FNA biopsy was considered unnecessary in these patients.

We compared the diagnostic efficiency of the two guidelines in our sample, irrespective of the nodules’ sizes. These values were also measured and compared exclusively in nodules with a diameter of >4 cm. The proportion of unnecessary FNA biopsies indicated by the two guidelines was then calculated and compared for all nodules. Unnecessary FNA was defined as an indication of biopsy in a benign lesion by each guideline.

### 2.4. Statistical Analysis

The distributions of the quantitative data were evaluated using the Kolmogorov–Smirnov test. Due to their abnormal distributions, these data (age, nodule size, and TSH levels) were presented as median and interquartile range (IQR). The Mann–Whitney U test was used to evaluate the relationship between these variables and malignancy. A chi-squared test of homogeneity was employed to examine the distribution of qualitative data (US characteristics) among malignant and benign nodules. The correlation of ACR TI-RADS and ATA guideline scores with malignancy was investigated using the Spearman rank test. Receiver operating characteristic (ROC) curve analysis was performed to establish the optimal cut-off point for each guideline to compare diagnostic performance. These ideal cut-off points were adopted to provide metrics for the two guidelines’ sensitivity, specificity, accuracy, negative predictive value (NPV), and positive predictive value (PPV) along with their confidence intervals. The accuracy, sensitivity, and specificity were compared between the two guidelines using the McNemar test. A comparison between the area under the curve (AUC) of the two guidelines was made using the DeLong et al. method [13]. In addition, to comprehensively compare the diagnostic performance of ATA and ACR TI-RADS, we conducted decision curve analysis (DCA). DCA combines the assessment of predictive performance with the clinical consequences of employing different diagnostic strategies. It allows for the determination of the net benefit associated with each guideline across a range of threshold probabilities. Multivariate logistic regression was employed to investigate the association of various demographic and US features with malignancy. All analyses regarding diagnostic performance (ROC curve, sensitivity, specificity, accuracy, PPV, and NPV) were conducted in MedCalc^®^ Statistical Software version 20.215. All other analyses were conducted in IBM SPSS statistics V 26, and R software (version 4.3.1, R Foundation for Statistical Computing, Vienna, Austria).

## 3. Ethical Considerations

The Ethical Committee of Mashhad University of Medical Sciences reviewed and fully approved this study under the code: IR.MUMS.fm.REC.1396.576. Patients’ privacy and confidentiality were respected along with the declaration of Helsinki, and all patients signed written consent.

## 4. Results

A total of 571 nodules were included in our study. The average age was 47.4 ± 14.3 and the majority of nodules occurred in females (91.1%). The malignancy rate of our study sample was 11.4%. Most of the malignant nodules (93.8%, *n* = 61) had post-surgery histopathologic results. Among malignant nodules, papillary thyroid carcinoma (PTC) was the most prevalent type of malignant lesion (78%). Follicular thyroid carcinoma (FTC) was identified in eight nodules, medullary thyroid cancer (MTC) in three nodules, and Hurthle cell carcinoma (HCC) in two nodules. Given the rarity of HCC, we describe the US characteristics of the two histopathologically diagnosed lesions: The first lesion was diagnosed in a 23-year-old male; upon US examination, a relatively large (largest diameter of 6.0 CMs) hyperechoic solid nodule with regular margin, oval (wider than tall) shape, central vascularity, and absence of any echogenic foci, was detected. The second lesion was seen in a 65-year-old female and was hypoechoic, solid, and taller than wide with microcalcification and peripheral vascularity. Similar to the first lesion, this nodule was relatively large, with a highest diameter of 6.7 CM. 

Table 1 illustrates the demographics and US characteristics of nodules. The distribution of margin (*p* < 0.001), echogenicity (*p* < 0.001), calcification (*p* < 0.001), shape (*p* < 0.001), and vascularity (*p* < 0.001) differed significantly between malignant and benign nodules, and a larger size was associated with malignancy (*p* < 0.001). Malignancy was more common in nodules with irregular margins, hypoechoic nodules, micro-calcified nodules, nodules that were taller than wide, and nodules that exhibited central vasculature.

### 4.1. Correlation of ACR TI-RADS and ATA Guidelines with Malignancy

As illustrated in Table 2, ACR TI-RADS and ATA guidelines were significantly correlated with malignancy (*p* < 0.01, *p* < 0.01, respectively). However, ACR TI-RADS was more closely correlated compared with the ATA guideline (R = 0.320 vs. R = 0.230). The distribution of malignant nodules among each category of ACR-TIRADS and ATA guideline also varied significantly (*p* < 0.05), increasing from TR1 and benign to TR5 and high suspicion, respectively.

### 4.2. ACR TI-RADS and ATA Guidelines Diagnostic Performance

The optimal cut-off values for all nodules and nodules larger than 4 cm were TR4 and TR5 for ACR-TIRADS, as well as intermediate and high suspicion for the ATA guideline (Table 3). ACR TI-RADS had an accuracy of 78.4% at these cut-off points across all nodules, regardless of size, whereas the ATA guideline had an accuracy of 80.9%. There was no statistically significant difference between these two guidelines’ accuracy (*p* = 0.203). Figure 1(B1,B2) illustrate a malignant nodule that was missed by both ACR-TI-RADS and the ATA guideline based on their optimal cut-off points.

**Figure 3:**
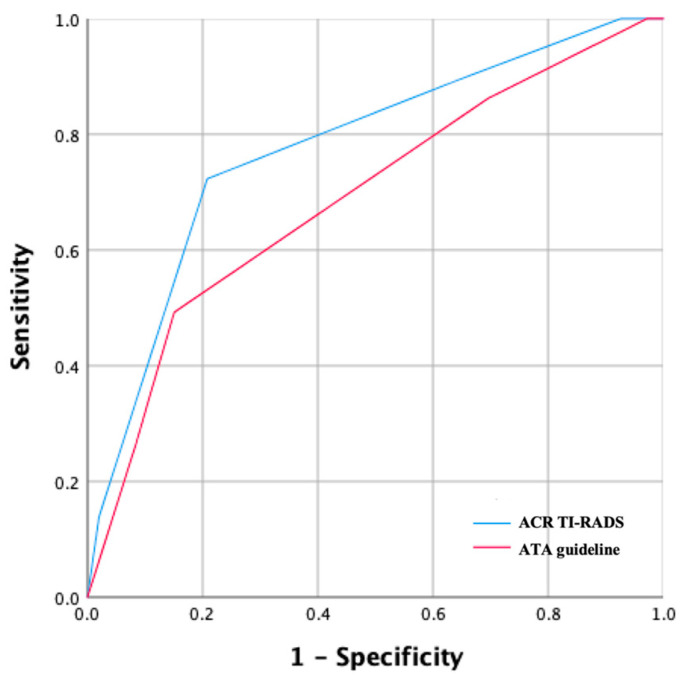
The ROC curve of the ATA guideline and ACR TI-RADS.

Figure 3 presents the ROC curves for the ATA guideline and ACR TI-RADS. The ATA guideline’s AUC was 0.691 (0.651–0.728), whereas the AUC for ACR TI-RADS was 0.776 (0.740–0.810). The ACR TI-RADS had a significantly greater AUC (*p* = 0.03) in comparison to the ATA guideline. The ACR TI-RADS was more sensitive (72.3 vs. 49.2, *p* = 0.003), while the ATA guideline was more specific (79.2 vs. 84.9, *p* < 0.001). Both guidelines had a considerably high NPV (95.7 for ACR TI-RADS and 92.8 for the ATA guideline), while the PPV was 30.9 for ACR TI-RADS and 29.6 for the ATA guideline.

In nodules larger than 4 cm, ACR TI-RADS had an accuracy of 78.4% and the ATA guideline’s accuracy was 80.5%, which were not statistically different (*p* = 0.885). Even though ACR TI-RADS had a greater AUC compared to the ATA guideline in nodules larger than 4 cm (0.806: 0.740–0.862 vs. 0.706: 0.632–0.772), this difference did not reach the conventional significance threshold (*p* = 0.09). In nodules larger than 4 cm, the sensitivity of ACR TI-RADS was significantly higher than the ATA guideline (78.4 vs. 43.2, *p* = 0.002) and the ATA guideline was more specific than ACR TI-RADS (78.8 vs. 89.8, *p* = 0.011).

**Figure 4:**
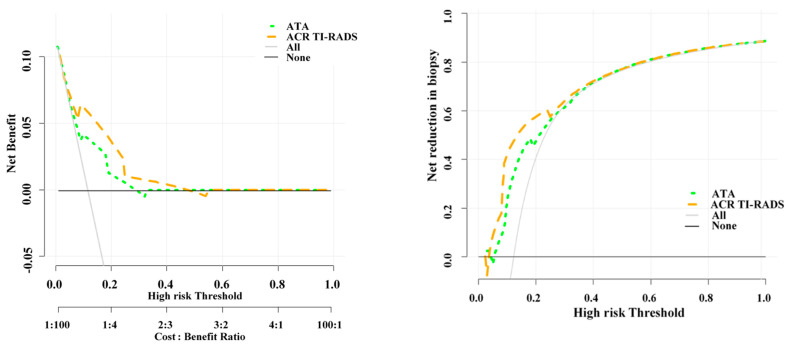
Decision curve analysis demonstrating the higher net benefit (**left**) and net reduction in biopsy (**right**) of ATA guideline and ACR TI-RADS in diagnosis of thyroid malignancy. In all ranges of threshold probability (i.e., 0.1 to 0.3), ACR TI-RADS outperformed ATA guidelines in detection of malignancy and reducing unnecessary biopsies.

The DCA results clearly demonstrated the superiority of ACR TI-RADS over ATA in terms of net benefit across a range of threshold probabilities (Figure 4 left). The curve for ACR TI-RADS consistently lies above the curve for ATA, indicating that ACR TI-RADS provides a higher net benefit in predicting thyroid malignancy. At a threshold probability of 10%, the net benefits of ACR TI-RADS and ATA guidelines are 0.062 and 0.041. It could be interpreted that compared to conducting no biopsies, obtaining biopsies on the basis of the ACR TI-RADS (ATA guideline) is equivalent to a strategy that identifies 6(4) cancers per hundred patients without conducting any unnecessary biopsies.

### 4.3. Unnecessary FNA Biopsy Rates of the Two Guidelines

The net reduction in unnecessary biopsy is higher for ACR TI-RADS than ATA guideline (Figure 4 right). At a probability threshold of 10%, the net reduction in biopsies based on the ACR TI-RADS and ATA guideline are about 41 and 23 per 100 patients. In other words, at this probability threshold, biopsying patients on the basis of the ACR TI-RADS (ATA guideline) is the equivalent of a strategy that reduced the biopsy rate by 41% (23%), without missing any cancers.

We followed our study sample for indication of FNA biopsy to determine the unnecessary FNA rates indicated by each guideline (Table 4). The total number of indicated biopsies and unnecessary FNA biopsy rates were lower in ACR TI-RADS than the ATA guideline. The ATA guideline had a higher detection rate than ACR TI-RADS (98.4% vs. 89.2%). The ACR TI-RADS presented no indication of biopsy in seven malignant nodules, all of which were categorized as TR2. As for the ATA guideline, only one malignant nodule was missed. Four of the seven nodules overlooked by ACR TI-RADS had a diameter of ≥4 cm. The other three ranged in size from 3 to 4 cm. Furthermore, three of the seven malignant nodules missed by the ACR TI-RADS exhibited central vascularity. We investigated the addition of the size (≥4 cm) and central vascularity criteria regarding FNA sampling for nodules categorized as TR2 since all missing malignant nodules were in this group. We observed a notable decrease in the number of missed malignant nodules (only two nodules would be missed using these criteria), while the total number of indicated FNA biopsies increased from 293 to 341.

### 4.4. Malignancy Predictors

The most prominent predictor of malignancy was the presence of hypoechogenicity in a nodule (Table 5), which raised the likelihood of malignancy by 8.34 times (OR = 8.34, 95% CI: 3.57–19.45). Other significant predictors of malignancy included taller-than-wide shape (OR = 6.73, 95% CI: 3.07–14.77), microcalcification (OR = 5.17, 95% CI: 2.10–12.69), male gender (OR = 3.35 95% CI: 1.34–8.36), central vascularity (OR = 2.93 95% CI: 1.35–6.38), and irregular margin (OR = 2.09 95% CI: 1.06–4.13). In our study sample, nodule size was also an important predictor of malignancy, with each 1 cm increase in the nodule’s greatest diameter associated with a 1.36 (95% CI: 1.14–1.61) increase in the probability of the nodule being malignant. 

## 5. Discussion

The ATA guideline and ACR TI-RADS are two of the most common risk stratification systems in evaluating thyroid nodules for malignancy [14]. Nevertheless, there are vast controversies regarding the diagnostic performance of these models, so we decided to conduct this study to evaluate the diagnostic performance of these guidelines in the Iranian population.

In the current study, we detected two lesions histopathologically diagnosed as Hurthle cell carcinoma (HCC). Given the rarity of this neoplasm, we chose to contribute to the literature by reporting and discussing the ultrasound characteristics of these nodules. Our findings align with previous research on Hurthle cell neoplasms. For instance, Kim et al. assessed 139 nodules histopathologically diagnosed as Hurthle cell neoplasms between 1996 and 2020 and found that tumor size was an independent predictor of malignancy within this group [15]. However, they did not observe an association between other suspicious ultrasound features and malignancy, their insights, combined with those of other groups, highlight the significance of nodule size in predicting malignancy in Hurthle cell neoplasms [16,17]. Our observations further affirm this trend. Both nodules diagnosed as HCC in our study had notably large diameters, measuring 6.0 cm and 6.7 cm, respectively. This consistency in findings across studies underscores the potential clinical value of incorporating nodule size into the assessment and management of Hurthle cell neoplasms. The collective body of evidence supports the need for continued investigation to enhance the understanding of ultrasound characteristics that contribute to the diagnosis and management of this rare but clinically important neoplasm.

The diagnostic performance of these two risk stratification systems has recently been assessed by two systematic studies; one concluded that neither of these guidelines is better than the other, while the other found that ACR TI-RADS is the most efficient risk stratification method [11,18]. In our study, the overall sensitivity of ACR TI-RADS was 72.3%, which was significantly higher than the ATA guideline’s sensitivity (49.2%, *p* = 0.003). Also, the ATA guideline appeared to be more specific compared to the ACR TI-RADS at their optimal cut-off points (79.2% vs. 84.9%, *p* < 0.001). The AUC was considerably higher in ACR TI-RADS, and the two guidelines did not show a significant difference concerning accuracy. ACR TI-RADS is more sensitive than the ATA guideline, meaning that a smaller number of malignant nodules will be missed in cases where clinical suspicion of malignancy is high in a nodule, whereas the ATA guideline’s higher specificity is helpful when a nodule is perceived as likely benign by a clinician.

As thyroid malignancy is more prevalent in nodules with a ≥4 cm diameter [19,20], we evaluated the diagnostic performance in these nodules exclusively. The ACR TI-RADS was also more sensitive than the ATA guideline for nodules with a diameter of ≥ 4 cm (*p* = 0.002). The sensitivity of ACR-TI-RADS increased from 72.3% for all nodules to 78.4% in nodules larger than 4 cm. The ATA guideline was also more specific in nodules with a diameter of ≥4 cm. This higher sensitivity rate in nodules with a diameter of ≥4 cm can help clinical decision-making, particularly when the FNA biopsy result is inconclusive.

Our findings revealed that the TR2 and low suspicion groups of these two risk stratification systems exhibited higher malignancy rates than the malignancy rates guidelines (6 vs. <3% for ATA guideline and 4.6 vs. 2%, for ACR TI-RADS) [3,6]. Huh et al. reported similar findings regarding ACR TI-RADS and ATA guideline ranks with low malignancy likelihood (TR2 and low suspicion) [21]. In line with our study, they also included patients for FNA sampling based on clinical gestalt; this increased incidence might be due to the unwarranted FNA biopsy in patients with thyroid nodules perceived benign by their normal US features, as they were not referred for FNA biopsy and therefore were not included in either study.

We also compared the efficiency of the FNA biopsy indications of the two guidelines in our study sample. The ACR TI-RADS had a lower unnecessary FNA rate and indicated a total number of 293 biopsies in our study sample, of which 80.2% were unnecessary. However, ACR TI-RADS FNA indication missed seven malignant nodules. The ATA guideline recommended FNA for 527 nodules, of which 87.8% were unnecessary and only one malignant lesion was missed. This notable difference between the two risk stratification systems regarding FNA indication may be due to the ACR TI-RADS greater size threshold in mildly suspicious nodules (TR3) and the elimination of FNA indication in nodules that are most likely benign (TR2). Since a small subset of malignant nodules displays nearly normal US characteristics, it is inevitable that ACR TI-RADS would miss these nodules. Four of the seven malignant nodules overlooked by ACR TI RADS were larger than 4 cm in diameter, while three exhibited central vascularity. The ACR TI-RADS gives no weight to the vascularity of nodules when determining their potential for malignancy. This approach also provides no FNA indication for nodules with minimal risk (TR2). As nodules’ vascularity and size were predictors of malignancy in our study and the available literature [22,23], we investigated supplementation of these two factors in the TR2 category and investigated whether a lower number of malignant nodules would be missed after these modifications. We established a criterion of >4 cm maximum diameter and central vascularity for FNA biopsies of TR2 nodules. The overall number of indicated biopsies would rise from 293 to 341, and five of these missed malignant nodules would not be overlooked. Determination of vascularity in thyroid nodules is highly affected by interobserver variability [24], and the decision to obtain an FNA biopsy based on these criteria should be made by experienced physicians in thyroid US examination. Similarly, Qiang et al. added vascularity to ACR-TIRADS FNA indications and observed a notable decrease (43%) in the number of missed malignant nodules [9]. Given the fact that the number of indicated FNAs after the addition of vascularity and size threshold to ACR TI-RADS is still lower than the ATA guidelines, this system effectively reduced the number of FNA biopsies in our study sample and can reduce health costs and patients’ discomfort. Aside from vascularity, and size, shear wave elastography (SWE) has been introduced into the evaluation of thyroid nodules in recent years [25]. Although these techniques are still not integrated into the present guidelines, their supplementation to various guidelines seems to improve the diagnosis of malignant nodules both in our study and in the available literature [26]. Further research is thus recommended on the supplementation of these techniques in nodules with nearly normal US characteristics.

Table 6 illustrates the most recent studies on the comparison of the ACR TI-RADS and ATA guidelines [7,8,9,10,21,27,28,29,30,31,32]. The sensitivity of ACR-TIRADS was reported between 38.8% and 100%, while its reported specificity ranged between 41%% and 93.2%. Moreover, ATA guideline’s sensitivity was reported as low as 23.3% and as high as 98.6%, while its reported specificity ranged between 11% and 84.4%. The vast differences in these studies are probably due to inter-observer variability, sample size, different reference tests for malignancy, and different malignancy rates among their samples. Moreover, each one of these studies has demonstrated a specific cut-off value for ACR-TIRADS and ATA guidelines. As anticipated, studies assessing these two guidelines at a cut-off point of FNA indication reported the lowest specificity for both guidelines [9,21,28]. This confirms our findings regarding the high amount of unnecessary indicated FNA biopsies of both guidelines. However, the higher reported specificities of ACR TI-RADS over ATA guidelines at this cut-off point confirms our findings regarding the lower proportion of unnecessary FNA biopsies for ACR-TIRADS. All studies with established cut-off points different from FNA indication have also reported lower unnecessary FNA rates in ACR-TIRADS [7,10,21,27,29].

Our findings revealed that ACR TI-RADS is more sensitive than ATA guidelines, while ATA is more specific. In line with our findings, Lin et al. reported a higher sensitivity in ACR TI-RADS, whereas ATA was more specific in their study [7]. Regarding their low reported sensitivity for both ACR TI-RADS and ATA guidelines (38.8% and 23.3%, respectively), it is worth mentioning that they assessed the diagnostic performance of these guidelines only on nodules with a definitive diagnosis of either follicular thyroid adenoma or FTC [7], while FTC was diagnosed only in eight nodules in our study.

Gacayan et al. have also stated findings similar to ours [30]. They considered FNA cytology as the reference for malignancy. However, they deemed Bethesda grade III as benign and grade IV as malignant [30]. Given the fact that malignancy is not always ruled out in grade III and not always present in grade IV Bethesda [33], these findings might be biased. Further research is thus beneficial in these types of nodules. Others including Koc et al. [10], Qiang et al. [9], and Thedinger et al. [28], included nodules with cytology reports of Bethesda grade III and IV in their analysis for sensitivity and specificity. This might be an important cause of the difference in their findings compared to ours.

In contrast to our findings, Qi et al. [29], and Zhang et al. [32], reported the ATA guideline as more sensitive and the ACR TI-RADS guideline as more specific. Their study samples consisted of smaller nodules than our sample, which might explain the contrasting results.

Steirfert et al. evaluated 1211 thyroid nodules and concluded that the ATA guideline was more sensitive, while there was no significant difference regarding specificity [8]. They only included indifferent or hypofunction nodules when assessed with thyroid scintigraphy, whereas we included all thyroid nodules regardless of their function. Other factors, including varying patient demographics, sample size, malignancy rate, and inter-observer variability, might have also played a role in the discrepancy between their findings and ours.

Our study had some limitations. First, patients were referred for FNA biopsy from different clinics, including otolaryngology, head and neck surgery, general surgery, and endocrinology clinics. Each practice might have its own decision-making criteria and therefore refer specific patients for further evaluation using FNA biopsy. Second, our study was conducted in only one tertiary hospital, which might have resulted in the homogeneity of our sample. Strengths of our study included its prospective modality, as ACR TI-RADS and ATA guideline scores were calculated prospectively at all stages under real-time US, which results in a more accurate calculation of these scores. Also, 61 out of 65 nodules deemed malignant had available post-surgical histopathologic results, which increased the confidence in our analysis.

## 6. Conclusions

In conclusion, the ATA guideline and ACR TI-RADS showed different advantages in terms of diagnostic performance for thyroid nodules’ evaluation. The ACR-TIRADS was more sensitive, whereas the ATA guideline was more specific at obtained cut-off points. The ACR TI-RADS had a lower unnecessary FNA rate and proposed a lower number of FNA indications in our study sample. However, a higher number of malignant nodules were missed by ACR TI-RADS. Since all nodules missed by ACR TI-RADS were classified as TR2, clinicians should consider further evaluating TR2 nodules for FNA sampling when using this guideline.

## Figures and Tables

**Table 1:** Demographic and ultrasonographic features.

Variables		Total (*n*: 571)	Benign (*n*: 506)	Malignant (*n*: 65)	Malignancy Rate (%)	*p*-Value
Age	Median (IQR)	48 (41–57)	48 (41–57)	45 (30–56)	-	0.197
	Range	17–86	18–86	20–84		
Gender	Male	51 (8.9)	41 (8.1)	10 (15.4)	19.6	0.053
	Female	520 (91.9)	465 (91.9)	55 (84.6)	9.4	
Size	Median (IQR)	3.0 (2.3–4.1)	3.0 (2.2–4.0)	4.0 (2.9–5.0)	-	<0.001
	Range	1.0–13.7	1.0–13.7	1.5–7.0		
TSH	Median (IQR)	2.0 (1.0–2.3)	2.0 (1.0–2.3)	2.0 (0.7–2.3)	-	0.992
	Range	0.3–15	0.5–14.8	0.3–15		
Composition	Predominantly cystic	41 (7.2)	41 (8.1)	0 (0)	0	0.165
	Spongiform	2 (0.4)	2 (0.4)	0 (0)	0	
	Mixed	198 (34.7)	182 (36)	16 (8.1)	8.1	
	Solid	330 (57.8)	281 (55.5)	49(75.4)	14.8	
Margin	Regular	418 (73.2)	387 (76.5)	31 (47.7)	7.4	<0.001
	Irregular	153 (26.8)	119 (23.5)	34 (52.3)	22.2	
Echogenicity	Anechoic	18 (3.2)	18 (3.6)	0 (0.0)	0	<0.001
	Isoechoic	445 (77.9)	410 (81)	35 (53.8)	7.9	
	Hypoechoic	49 (8.6)	27 (5.3)	22 (33.8)	44.9	
	Hyperechoic	59 (10.3)	51 (10.1)	8 (12.3)	13.6	
Calcifications	None	489 (85.6)	440 (87)	49 (75.4)	10	<0.001
	Microcalcification	39 (6.8)	26 (5.1)	13 (20)	33.3	
	Macrocalcification	43 (7.5)	40 (7.9)	3 (4.6)	7	
Shape	Taller-than-wide	59 (10.3)	38 (7.5)	21 (32.3)	35.6	<0.001
	Wider-than-tall	512 (89.7)	468 (92.5)	44 (67.7)	8.6	
Vascularity	Central	63 (11)	43 (8.5)	20 (30.8)	31.7	<0.001
	Peripheral	508 (89)	463 (91.5)	45 (69.2)	11.4	

**Table 2:** Correlation of ACR TI-RADS and ATA guideline with malignancy.

Scoring System and Category		Benign *n* (%)	Malignant *n* (%)	Malignancy Rate (%)	Suggested Risk of Malignancy	*p*-Value (R)
ACR TI-RADS	TR1	38 (7.5)	0 (0.0)	0.0	0–2%	<0.01 (0.320)
	TR2	145 (28.7)	7 (10.8)	4.6	0–2%	
	TR3	218 (43.1)	11 (16.9)	4.8	5%	
	TR4	95 (18.8)	38 (58.5)	31.7	5–20%	
	TR5	10 (2)	9 (13.8)	47.4	>20%	
ATA guideline	Benign	14 (2.8)	0 (0)	0	<1%	<0.01 (0.230)
	Very low suspicion	140(27.7)	9 (13.8)	6	<3%	
	Low suspicion	275 (54.5)	24 (36.9)	8	5–10%	
	Intermediate suspicion	34 (6.7)	15 (23.1)	30.6	10–20%	
	High suspicion	42(8.3)	17 (26.2)	28.8	>70%	

**Table 3:** Diagnostic performance of ACR-TIRADS, and ATA-2015 guidelines.

Scoring System		Cut-Off	Accuracy (95% CI)	AUC (95% CI)	Sensitivity (95% CI)	Specificity (95% CI)	PPV (95% CI)	NPV (95% CI)
Overall *n* = 571	ACR TI-RADS	TR4/TR5	78.4 (74.8–81.76)	0.776 (0.740–0.810)	72.3 (59.8–82.7)	79.2 (75.4–82.7)	30.9 (26.3–36)	95.7 (93.7–97)
	ATA guideline	Intermediate/high suspicion	80.9 (77.4–84.0)	0.691 (0.651–0.728)	49.2 (36.6–61.9)	84.9 (81.5–87.9)	29.6 (23.4–36.7)	92.8 (91.1–94.3)
*p*-value			0.203	0.03	0.003	< 0.001	-	-
Nodules ≥ 4 cm *n* = 169	ACR TI-RADS	TR4/TR5	78.7 (71.7–84.6)	0.806 (0.740–0.862)	78.4 (61.8–90.2)	78.8 (71.0–85.3)	50.0 (40.1–59.0)	93.1 (87.9–96.1)
	ATA guideline	Intermediate/high suspicion	80.5 (73.7–86.2)	0.706 (0.632–0.772)	43.2 (27.1–60.5)	89.8 (83.4–94.3)	53.3 (38.1–68.0)	84.5 (81.5–88.6)
*p*-value			0.885	0.09	0.002	0.011	-	-

**Table 4:** Comparison of unnecessary FNAB rates and number of missed malignant nodules in ACR TI-RADS and ATA guideline.

Scoring System		No. of Indicated Biopsies	No. of Malignant Nodules among FNAB Indicated Nodules	Unnecessary FNAB Rate	No. of Missed Malignant Nodules	Detection Rate
ACR TI-RADS	Total	293	58	80.2%	7	89.2%
	TR5	19	9	52.6%	0	100%
	TR4	116	38	67.2%	0	100%
	TR3	158	11	93%	0	100%
	TR2	0	0	-	7	0%
ATA Guideline	Total	527	64	87.8%	1	98.4%
	High suspicion	59	17	71.2%	0	100%
	Intermediate suspicion	49	15	69.4%	0	100%
	Low suspicion	291	24	91.8%	0	100%
	Very low suspicion	128	8	93.7%	1	90%

**Table 5:** Association of demographic features and US characteristics with malignancy.

Parameters	Univariate Analysis (Crude)				Multivariate Analysis (Adjusted) *			
	β	SE	OR	*p*-Value	β	SE	OR	*p*-Value
Microcalcification	1.50	0.37	4.48 (2.16–9.30)	<0.001	1.64	0.45	5.17 (2.10–12.69)	<0.001
Hypoechogenicity	2.25	0.33	9.54 (4.93–18.47)	<0.001	2.12	0.43	8.34 (3.57–19.45)	<0.001
Irregular margin	1.27	0.26	3.56 (2.10–6.04)	<0.001	0.74	0.34	2.09 (1.06–4.13)	0.032
Taller-than-wide shape	1.77	0.31	5.87 (3.17–10.88)	<0.001	1.90	0.40	6.73 (3.07–14.77)	<0.001
Central vascularity	1.56	0.30	4.78 (2.58–8.82)	<0.001	1.07	0.39	2.93 (1.35–6.38)	0.006
Size	0.32	0.08	1.39 (1.18–1.63)	<0.001	0.31	0.08	1.36 (1.14–1.61)	<0.001
Male gender	0.72	0.38	2.06 (0.978–4.34)	0.057	1.20	0.46	3.35 (1.34–8.36)	<0.009

* Multivariate model was adjusted for age, and serum TSH levels as continuous variables, and all collected US features and gender as categorical variables.

**Table 6:** Recent studies on the evaluation of ACR TI-RADS and ATA guidelines.

Study (Year)	No. of Nodules	Age	Gender (F/M)	Mean Nodule’s Size	Optimal Cut-Off Value		Sensitivity (%)		Specificity (%)		AUC		Unnecessary FNA Rate (%)		Reference Test
					ATA Guideline	ACR TI-RADS	ATA Guideline	ACR TI-RADS	ATA Guideline	ACR TI-RADS	ATA Guideline	ACR TI-RADS	ATA Guideline	ACR TI-RADS	
Thedinger et al. (2022) [28]	236	58.7 ± 0.6	538/182	-	Indication of FNA by guideline	FNA indication	81.6	73.7	54.5	27.0	-	-	72.3	82.3	Cytology
Qi et al. (2022) [29]	820	44.5 ± 13.4	619/201	13 mm	High suspicion	TR5	92.1	79.3	88.3	93.2	0.921	0.925	47.4	26.3	Cytology or pathology
Lin et al. (2022) [7]	455	43.5 ± 14.3	216/113	3.6 ± 1.7 cm	High and intermediate suspicion	TR4-TR5	23.3	38.8	87.7	80.2	0.555	0.595	90.5	65.3	Pathology
Gacayan et al. (2021) [30]	197	53 (21–77)	103/18	-	High and intermediate suspicion	TR4-TR5	88.2	100	57.8	52.2	-	-	-	-	Cytology
Qiang et al. (2021) [9]	417	58.7 ± 14	334/83	-	Indication of FNA by guideline	FNA indication	97	70	11	29	-	-			Cytology or pathology
Zhu et al. (2021) [31]	2309	53.2 ± 12.7	1336/361	13.1 ± 10.6 mm	High suspicion	TR5	96.51	93.71	67.1	74.82	0.824	0.854	29.41	29.41	Cytology or pathology
Zhang et al. (2021) [32]	566	47.4 ± 13.4	442/124	11.4 ± 5.8 mm	High suspicion	TR5	89.7	86.5	84.4	88.4	0.907	0.894	-	-	Cytology or pathology
Seifert et al. (2021) [8]	1211	51 ± 14	604/249	26 ± 13 mm	High suspicion	TR5	77	68	80	80	0.795	0.801	-	-	Cytology or pathology
Huh et al. (2021) [21]	1384	50.2 ± 13.6	1062/239	23.2 ± 12.6 mm	Indication of FNA by guideline	FNA indication	98.6	80.4	19.9	62.2	0.592	0.713	75.3	63.8	Cytology or pathology
Chen et al. (2022) [27]	146	56.7	99/26	median: 2.3 cm	High and intermediate suspicion	TR4-TR5	91	75	26	41	-	-	68.5	54.8	Cytology or pathology
Koc et al. (2020) [10]	492	52 (18–84)	375/85	-	High and intermediate suspicion	TR4-TR5	82.22	48.89	53.47	60.63	0.740	0.550	88.8	80.8	Cytology or pathology

## Data Availability

The data supporting this study’s findings are available from the corresponding author upon reasonable request.
